# Advancing the science of health professions education through a shared understanding of terminology: a content analysis of terms for “faculty”

**DOI:** 10.1007/s40037-021-00683-8

**Published:** 2021-09-10

**Authors:** Pim W. Teunissen, Anique Atherley, Jennifer J. Cleland, Eric Holmboe, Wendy C. Y. Hu, Steven J. Durning, Hiroshi Nishigori, Dujeepa D. Samarasekera, Lambert Schuwirth, Susan van Schalkwyk, Lauren A. Maggio

**Affiliations:** 1 Maastricht University Medical Center, Maastricht, The Netherlands; 2 School of Health Professions Education (SHE), Faculty of Health Medicine and Life Sciences, Maastricht University, Maastricht, The Netherlands; 3 Western Sydney University, Sydney, Australia; 4 LKC School of Medicine, Nanyang University Singapore, Singapore, Singapore; 5 University of Aberdeen, Aberdeen, UK; 6 Accreditation Council for Graduate Medical Education, Chicago, IL USA; 7 Center for Health Professions Education and Department of Medicine, Uniformed Services University of the Health Sciences, Bethesda, MD USA; 8 Center for Medical Education, Nagoya University, Nagoya, Japan; 9 Centre for Medical Education (CenMED), National University of Singapore, Singapore, Singapore; 10 Ministry of Health Singapore, Singapore, Singapore; 11 Flinders Health and Medical Research Institute, Prideaux at Flinders University, Adelaide, Australia; 12 Centre for Health Professions Education and Faculty of Medicine and Health Sciences, Stellenbosch University, Stellenbosch, South Africa

**Keywords:** Faculty terminology, Literature study, Content analysis

## Abstract

**Introduction:**

Health professions educators risk misunderstandings where terms and concepts are not clearly defined, hampering the field’s progress. This risk is especially pronounced with ambiguity in describing roles. This study explores the variety of terms used by researchers and educators to describe “faculty”, with the aim to facilitate definitional clarity, and create a shared terminology and approach to describing this term.

**Methods:**

The authors analyzed journal article abstracts to identify the specific words and phrases used to describe individuals or groups of people referred to as faculty. To identify abstracts, PubMed articles indexed with the Medical Subject Heading “faculty” published between 2007 and 2017 were retrieved. Authors iteratively extracted data and used content analysis to identify patterns and themes.

**Results:**

A total of 5,436 citations were retrieved, of which 3,354 were deemed eligible. Based on a sample of 594 abstracts (17.7%), we found 279 unique terms. The most commonly used terms accounted for approximately one-third of the sample and included faculty or faculty member/s (*n* = 252; 26.4%); teacher/s (*n* = 59; 6.2%) and medical educator/s (*n* = 26; 2.7%) were also well represented. Content analysis highlighted that the different descriptors authors used referred to four role types: healthcare (e.g., doctor, physician), education (e.g., educator, teacher), academia (e.g., professor), and/or relationship to the learner (e.g., mentor).

**Discussion:**

Faculty are described using a wide variety of terms, which can be linked to four role descriptions. The authors propose a template for researchers and educators who want to refer to faculty in their papers. This is important to advance the field and increase readers’ assessment of transferability.

**Supplementary Information:**

The online version of this article (10.1007/s40037-021-00683-8) contains supplementary material, which is available to authorized users.

## Introduction

Without definitional clarity of terms and concepts, researchers and educators in health professions education (HPE) research risk misunderstandings, slow the progress of the field, and limit the possibility for newcomers to engage in scholarly conversations [1]. In 2021, this warning still resonates. Clarity or a shared understanding of terminology remains elusive across HPE. For example, HPE authors have published on variability in defining key concepts (e.g., teaching tools, competency-based medical education) within the published literature [2, 3] and definitional differences even within research teams [2–4]. These authors also highlighted that this lack of agreement can contribute to miscommunication, lead to suboptimal research on a topic, and complicate the researchers’ ability to build on previous research. Within our diverse author team, we arrived at a similar realization.

Our observation of definitional disharmony stemmed not from a particular topic, but rather related to the multiple terms that are used to describe people engaged in training health professionals and those conducting HPE research. Our conversations indicated that this creates and replicates misunderstandings that ripple across many topics and concepts. For example, regarding terms related to what may be colloquially known as faculty, our European authors were unfamiliar with the term “attending physician”, whereas those of us in North America were unfamiliar with the term “specialist registrar”. Thus, this current study investigates varieties of faculty terminology, which we defined for the purposes of this study as a person (or group of people) who engage in activities, such as teaching, assessment, mentoring, and/or supervision, intended to contribute to the education of learners in the health professions.

While faculty is a common name for this, it takes little imagination to come up with other terms—clinical supervisor, teacher, preceptor, attending physician, or clinical staff—to describe the same (groups of) individuals. As a result, a study on workplace-based teachers in an undergraduate medical program and another study on attending physicians might appear to study different groups, and yet, despite using different terms, a careful examination of the population description might reveal workplace-based teachers in an undergraduate program to be attending physicians at an affiliated hospital. Alternatively, two studies focusing on teach-the-teacher programs might seem related. However, closer inspection could show faculty in one study focused on biomedical scientists while another examined nurse practitioners. Our field’s diversity of research participants, combined with a lack of clear guidance on what is helpful in describing one’s research participant pool, inhibits our ability to build on previous work, contribute to the development of research-informed theory, and provide optimal educational practice.

In this study, we have three aims: 1) to analyze and report the terminology used to describe “faculty” throughout the biomedical literature (including HPE papers), 2) to offer guidance on key aspects, in template form, that researchers and educations should include when describing one’s research participants in scientific publications, and 3) to provide a replicable study method that can be utilized to explore other key terms in HPE (e.g., to unpack those who we colloquially call medical students and residents).

## Methods

We conducted a content analysis of journal article abstracts to identify the presence of specific words used to describe individuals or groups of people referred to as faculty.

To provide a variety of geographical and cultural perspectives on the term “faculty”, we assembled a global author team with diverse backgrounds (for the distribution of the research team see Fig. S1 in Electronic Supplementary Material [ESM]). Due to authors’ diverse geographical locations, the team met in-person in 2018 and 2019 at the annual meeting of the Association of Medical Educators of Europe (AMEE) and held team meetings via teleconferencing software to provide updates on the project and receive feedback on the coding and analysis of the data.

We searched PubMed on September 11, 2018, for articles published between 2007 and 2017 that were indexed with the Medical Subject Heading (MeSH) “faculty” and related, more specific MeSH terms including “faculty, dental”, “faculty, medical”, “faculty, nursing”, and “faculty, pharmacy” (see the Appendix in the Electronic Supplementary Material [ESM] for our search strategy). The National Library of Medicine defines faculty as “teaching and administrative staff having academic rank in a post-secondary educational institution” [5]. We decided to use MeSH terms that would provide a wide variation of papers from our search, but that were determined by professional indexers to be about “faculty”. We acknowledge that this search does not provide a complete overview of all papers that research faculty or that mentioned participants that are relevant to our aims. We did not limit our search based on language to ensure we captured global perspectives but did restrain it to the last decade to ensure that the terminology in our data was recent. We utilized PubMed, a freely available database, as our only database to enable future researchers to replicate our method without database access concerns. Additionally, we focused solely on PubMed, in order to take advantage of the consistency of MeSH indexing, which is generated from a single, consistent entity (i.e., the National Library of Medicine).

We downloaded the citations retrieved from our search with the accompanying metadata into Excel for data management. Metadata included the abstract text (all of which is in English), publication year, journal, language, and the first author’s location. LM screened all titles and abstracts to exclude citations without abstracts and those that were historical descriptions of faculty members.

The search retrieved 5,436 citations, of which we excluded 2,082 articles that did not include abstracts or were of a historical nature. Thus, our final sample included 3,354 citations. We took an iterative data extraction approach, which focused on the abstract text. To begin, PT and LM devised a broad working definition for faculty following an initial abstract review and discussion. PT and LM examined the abstract text of 35 randomly selected citations and extracted from each abstract terms and brief descriptions that referred to faculty. They discussed their findings. Based on this preliminary data extraction, we developed an initial working definition to guide our teams’ process of then extracting “faculty” terms from the abstracts. PT and LM defined faculty as:* “a person or group of people who engage in some sort of activity that is intended to impact the development of another person or group”*. Using this working definition, seven project team members (PT, LM, AA, SD, WH, JC, SvS) independently extracted faculty terms from 144 randomly selected, unique abstracts. Additionally, to calibrate our data extraction, team members examined 10 of the same abstracts, resulting in data extracted from a total of 154 abstracts.

PT, LM, and AA met via conference call to discuss the results of the group’s term extraction and feedback on the working guideline. PT, LM, and AA individually developed and then discussed interim interpretations. Next, all team members were again invited to extract terms using these revised guidelines (e.g., code abstract as “null” if it should be excluded with an explanation, such as: a paper describing a personal history or one describing an organizational unit as faculty [no research participants]). Ten project team members (PT, LM, AA, SD, JC, LS, HN, DS, EH, and SvS) and one other person (a research assistant working with SvS) independently extracted terms from 40 randomly selected, unique abstracts resulting in 440 coded abstracts. Thus, in combination with earlier efforts, the authors had now extracted 279 unique terms for faculty from 594 abstracts, which represented 17.7% of the total number of abstracts in our sample (*n* = 3,354). We stopped at this point as we noticed that the final coding round did not yield any major alterations in our working categories and analysis output. We identified 106 abstracts as “null” (17.8%).

We used Google Sheet to organize and facilitate the analysis, including the calculation of descriptive statistics. We assigned each abstract to a geographical region to categorize the first authors’ location.

We used content analysis to identify patterns and themes in the extracted terms. Content analysis is described as a *“qualitative data reduction and sense making effort that takes a volume of qualitative material and attempts to identify core consistencies and meanings” *[6, p. 541]. To start, PT, AA, and LM independently read all extracted terms multiple times to identify patterns or themes. Preliminary themes were then discussed collectively in three teleconference calls, which resulted in a working framework for organizing the terms. PT presented this framework to the author team via conference call and an in-person meeting at an international conference to ensure that the framework resonated with the author team’s experience of data extraction and their cultural perspectives. No significant changes were necessary.

## Results

Abstracts were written by first authors representing 54 of 93 countries in the 3,354 articles initially extracted. The articles in our sample were predominantly written in English (90.9%) and published in 260 journals. Of the 594 abstracts coded, the majority (*n* = 346) were published by first authors based in North America (58.25%) (see Table S1 in Electronic Supplementary Material [ESM]), of which 305 were located in the United States and Canada (*n* = 41).

Authors described “faculty” using 279 unique terms coded a total of 954 times in the 594 abstracts. Of these 954, the most frequently used terms included faculty or faculty member/s (*n* = 252; 26.4%); see Tab. 1. For example, one study described its purpose as: *“To assess knowledge and perceptions of plagiarism in medical students and* *faculty of private and public medical colleges in Karachi”* [7, underlining added]. Additionally, [adjective] faculty or faculty member/s was coded 71 times (e.g., [clinical] faculty). Together, faculty (including faculty member/s) and [adjective] faculty (including faculty member/s) accounted for 33.8% of all codes. Codes for teacher/s (*n* = 59; 6.2%) and medical educator/s (*n* = 26; 2.7%) were well represented, accounting for almost 10% of all codes.

**Table 1:** Top 10 terms used for faculty by number of codes

Term	Number of codes (%)
Faculty or Faculty member/s	252 (26.4)
[Adjective]^a^ faculty	71 (7.4)
Teacher/s	59 (6.2)
Medical educator/s	26 (2.7)
Educator/s	25 (2.6)
Physician/s	16 (1.7)
Preceptor	16 (1.7)
Clinical Teacher/s	13 (1.4)
Professor/s	13 (1.4)
Supervisor/s^b^	11 (1.2)
Trainer/s^b^	11 (1.2)

^a^Examples here include junior faculty, full-time faculty, university faculty, emergency medicine faculty

^b^Eleven terms have been included in this table, since there was a tie for 10th place (at 11 each)

Similar to the overall sample, we observed some level of variation in the terms used by authors across geographical regions (see Appendix in ESM). For example, the top term used in Europe & Central Asia and Latin America and the Caribbean was “teachers”, whereas, in all other regions, the top term was faculty/faculty members. Notably, we could not determine top terms in sub-Saharan Africa as all ten terms were coded once in abstracts from that region.

Forty-four percent of abstracts referred to the study population using more than one term. For example, one abstract described a study to understand participants’ conceptions of teaching and learning and labeled the participants as “medical teachers”, “teachers”, and “faculty”. On average, each abstract was coded with 1.74 terms related to faculty with a range of 1–6 terms for any single abstract. Notably, we observed that even in publications on the same topic, authors used different terms to describe faculty populations. For example, in three abstracts focused on professionalism we observed the use of: medical teachers [8], physicians [9], and faculty [10].

Based on our content analysis of the abstracts and identified terms, we observed that authors referred to faculty based on role types. We identified four types of roles: roles in healthcare *(e.g., doctor, physician)*, roles in education *(e.g., educator, teacher)*, roles in academia *(e.g., faculty member, professor),* or roles in relationship to the learner *(e.g., mentor, supervisor); *see Fig. S2 in ESM and, for a list of all terms extracted, see the Appendix in ESM.

In addition to using multiple terms to describe faculty, we identified instances in which a single abstract included terms that cut across our role categories. For example, one abstract reads as follows: “… *physicians involved in medical teaching at Göttingen Medical School, Germany, were invited to complete an online survey addressing their views on clinical teachers’ educational skills. In addition, physicians’ motivation to engage in pedagogical training was assessed*” [11, underlining added]. This example uses terms that describe participants’ roles in both health care and education to name the same group of individuals imparting knowledge or skills to learners.

## Discussion

We identified 279 unique terms used to describe a person or group of people who engage in some sort of activity intended to impact another person or group’s development. These ranged from faculty to teacher to trainer. We were encouraged that the varied use of terms could be mapped to one of four categories related to role type: role in healthcare, role in education, role in academia, or relationship to learner. We also observed that many abstracts used more than one term, or reference to more than one role, when describing the same group in the same study.

The high level of variation we observed aligns with findings of related studies focused on definitional clarity related to specific topics [2, 3]. For example, researchers studying definitions of competency-based medical education identified 19 definitions in a sample of 80 articles [2]. Our findings also align with research by Varpio and colleagues, who encountered a problem of differing terminologies describing HPE scholarship roles and organizational structures [12]. We observed cultural variation in terms used, though we saw similarities across world regions regarding terms used most frequently; one must accept that these terms could be used to mean different things in these countries.

In some countries where English is not the native language, the term “faculty” is used in a different way to describe the same concept. In Japan, for example, the words “Shidoi” and “Kyoin” are used in a similar sense to “faculty”, but they have different meanings due to cultural differences. If international joint research on the definition of “faculty” is to be further developed, it is necessary to investigate the use of different words to describe the same concept across world regions.

### Implications for theory building and collaboration in medical education

Misunderstandings affecting communication could be due to a lack of a shared understanding on related concepts and terminology in intercultural collaboration settings [13]. When researchers and educators use terms in varied ways, this potentiates misunderstanding and hampers progress and reader/researcher engagement in the field. Thus, it becomes difficult to build on existing research due to a lack of clear terminology. This threatens international collaborations. If we want to advance HPE with international research teams and enable them to jointly build theory, it is critical that we find ways of understanding how different terms may mean different things in varied contexts. To achieve this, authors need to better articulate theories, reasoning and values to facilitate collaboration and mitigate issues arising from the many academic traditions held by HPE researchers [14]. Further, theoretical frameworks should be increasingly incorporated in research, and we should build more on previous research [15]. This is difficult to do without definitional clarity, but as a field we are making progress. For example, scholars have recently explored how social learning theory has been used as a starting point to advance knowledge and further guide practice in health professions education [16]. With this present study, we too contribute to these thoughts on advancing knowledge, guiding practice, and facilitating collaboration through language. A shared language describing not only processes and outcomes but also the groups within HPE research is necessary.

Just as we strive for shared understanding related to theories, the terms we use to describe our teachers and students must facilitate global understanding, so we can accurately interpret each other’s work. An often-neglected prerequisite for building on each other’s research findings and developing a shared understanding of phenomena of interest to health professions education is recognizing who has studied similar phenomena. We now offer readers a template to describe a research population that includes people colloquially known as “faculty” in some regions.

### Template proposal

**Fig. 1:**
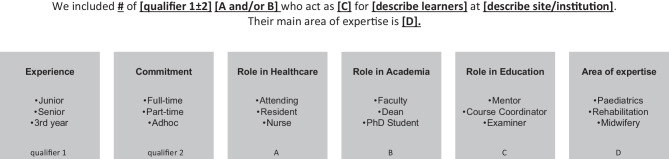
A template for describing research participants. Nota bene—Learners can be: medical students, clinical students, residents, lecturers. Site/institution can be: University name, clerkship location (e.g. ward, operating theatre)

Medical education has been described as a “melting pot”, with researchers hailing from a variety of academic traditions [14]. Adding this “pot” to cultural and regional variation, true shared understanding may be difficult. Thus, in an attempt to bring our field closer to a shared approach to describing faculty or other populations in HPE, we present a template (Fig. 1), which we propose researchers and educators can use when describing participants in a faculty-related role. This template maintains the existing practice we saw of using a term to describe research participants and another which further qualifies that term (see Fig. 1). We hope it will provide a structure to describe research participants not only in the body of articles, but also in abstracts. Additionally, we propose this clear description could assist future collaboration and interpretation of literature in HPE. We believe being explicit about who research participants are, what their role is, and their level/area of expertise is crucial to individual interpretation. It will also facilitate researchers building on previous studies, thereby growing the body of knowledge in a particular field. Single definitions of every unique term that can be used to describe those who are colloquially known as “faculty” will be difficult. Instead, we suggest explicit descriptions of research participants to minimize confusion.

Based on this template (Fig. 1), a completed example in the methods section of a paper might read as follows:

We included 711 full-time physicians who act as course coordinators for third year medical students at The University of The West Indies. These physicians are from varied specialties including paediatrics, surgery, and medicine.

Using the full template may not always be possible or practical, and it can be adapted for specific needs. For instance, in an abstract, one might use only the first sentence from the example and leave further details for the methods section. Research participants are also often described in the reporting of research questions. Using the template, a research question could be described as follows: *“Thus, this study aimed to answer the following research question: How do* *junior clinician educators view their roles in teaching at the bedside?”* As this example shows, the suggested template aims to clearly report information related to the research participants’ role in healthcare, their role in education, their role in academia, and their relationship to learners.

### Strengths and limitations

In our approach, we systematically identified abstracts based on how the United States (US) National Library of Medicine indexed articles with the MeSH “faculty”. We recognize that the US-based indexers may have inadvertently introduced their own cultural biases into their work. In our coding, we identified 106 abstracts as “null” or without terminology related to faculty. Taken together, this suggests that there may be some inconsistencies in indexing. To gather our sample, we purposely chose PubMed as it is a freely available resource. However, it does not include coverage of all medical education journals and thus of journals that might be influential in specific countries or regions, an issue not unique to PubMed.

As we used only (parts of) publications in English, we have to acknowledge that authors from non-English backgrounds had to translate their native language concepts into English. This is often more than merely a translation and may involve a trans- or even deculturation. In that case, our content analysis may contain incomplete domains and examples, but it reinforces our plea to use a template for the description of research participants rather than a single word.

Furthermore, we were unable to determine how many papers in medical education literature in non-English speaking countries use words similar to “faculty”, what other terms are used, and how they may add meanings that are not captured in English. Research focusing on terms related to faculty in non-English speaking countries needs to be carried out in the future as part of international joint research.

## Conclusion

We identified and described the varying use of faculty-related terms in almost 600 abstracts from biomedical literature, including that covering HPE. We mapped the terms to four key faculty roles and used them to offer researchers a template for use when describing research participants. This is a step toward shared understanding of commonly used terms in HPE. We invite other researchers to adopt our methodology to investigate the extent of variation in other terms within HPE, and to use and test our template.

## Supplementary Information

Fig. S1 Global distribution of our research team. Nota bene: Notably, Jennifer Cleland is Scottish but now works at an Asian institution; she started this project while still in Scotland and thus has markers in both locations. All location markers are approximations

Fig. S2 Selected examples of faculty-related terms as organized by observed faculty roles

Appendix

Table S1 Geographical distribution of first author locations according to region
