# Supplementary figures and images for: Advancing the science of health professions education through a shared understanding of terminology: a content analysis of terms for “faculty”
Source: Perspect Med Educ. 2021 Sep 10;11(1):22–7. doi: 10.1007/s40037-021-00683-8 (PMC8733114; doi:10.1007/s40037-021-00683-8)

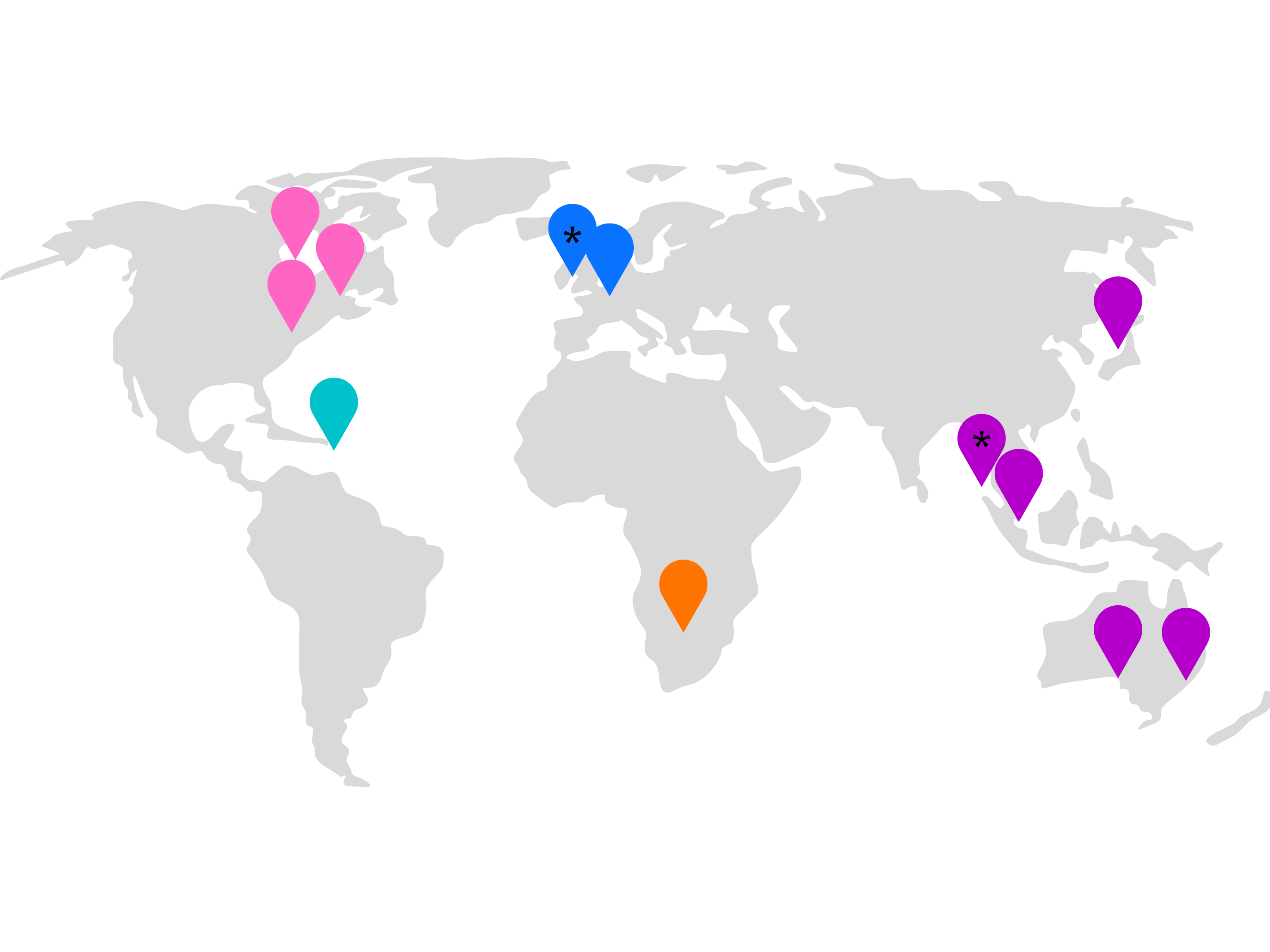
**Fig. 1**

Supplement: Supplementary file 1 — Fig. S1 Global distribution of our research team. Nota bene: Notably, Jennifer Cleland is Scottish but now works at an Asian institution; she started this project while still in Scotland and thus has markers in both locations. All location markers are approximations [file 40037_2021_683_MOESM1_ESM.docx]
