# Supplementary material for: Advancing the science of health professions education through a shared understanding of terminology: a content analysis of terms for “faculty”
Source: Perspect Med Educ. 2021 Sep 10;11(1):22–7. doi: 10.1007/s40037-021-00683-8 (PMC8733114; doi:10.1007/s40037-021-00683-8)
Supplement: Supplementary file 2 — Fig. S2 Selected examples of faculty-related terms as organized by observed faculty roles [file 40037_2021_683_MOESM2_ESM.docx]

Figure 2

FACULTY
