# Supplementary material for: Advancing the science of health professions education through a shared understanding of terminology: a content analysis of terms for “faculty”
Source: Perspect Med Educ. 2021 Sep 10;11(1):22–7. doi: 10.1007/s40037-021-00683-8 (PMC8733114; doi:10.1007/s40037-021-00683-8)
Supplement: Supplementary file 3 — Appendix [file 40037_2021_683_MOESM3_ESM.docx]

Strategy terms used to search PubMed on September 11, 2018 limited to 2007-2017

| **Search Terms** | **Indexing** |
| --- | --- |
| Faculty | MeSH |
| Faculty, Dental | MeSH |
| Faculty, Medical | MeSH |
| Faculty, Nursing | MeSH |
| Faculty, Pharmacy | MeSH |

Search strategy:

Faculty[MeSH] OR Faculty, Dental[MeSH] OR Faculty, Medical[MeSH] OR Faculty, Nursing[MeSH] OR Faculty, Nursing[MeSH] OR Faculty, Pharmacy[MeSH]

Notes:

MeSH=medical subject headings
