# Supplementary material for: Advancing the science of health professions education through a shared understanding of terminology: a content analysis of terms for “faculty”
Source: Perspect Med Educ. 2021 Sep 10;11(1):22–7. doi: 10.1007/s40037-021-00683-8 (PMC8733114; doi:10.1007/s40037-021-00683-8)
Supplement: Supplementary file 4 — Table S1 Geographical distribution of first author locations according to region [file 40037_2021_683_MOESM4_ESM.docx]

**Table 1** Geographical distribution of first author locations according to region

| **Region** | **All abstracts (*n*)** | **All abstracts**  **(%)** | **Coded abstracts**  **(*n*)** | **Coded abstracts (%)** |
| --- | --- | --- | --- | --- |
|  |  |  |  |  |
| North America | 2217 | 66.1% | 346 | 58.2% |
| Europe and Central Asia | 625 | 18.6% | 135 | 22.7% |
| East Asia and Pacific | 204 | 6.1% | 36 | 6.1% |
| South Asia | 104 | 3.1% | 20 | 3.4% |
| Middle East and North Africa | 88 | 2.6% | 32 | 5.4% |
| Latin America and the Caribbean | 75 | 2.2% | 16 | 2.7% |
| Sub-Saharan Africa | 41 | 1.2% | 9 | 1.5% |
